# The evaluation of IgG4 and IgG expression in cutaneous Rosai-Dorfman disease^⋆^

**DOI:** 10.1016/j.abd.2022.07.010

**Published:** 2023-06-08

**Authors:** Puyu Zou, Yi Zhan, Ruzeng Xue, Yu Liu, Guiying Zhang

**Affiliations:** a Department of Dermatology, Second Xiangya Hospital, Central South University, Changsha, China; b Dermatology Department, Dermatology Hospital, Southern Medical University, Guangzhou, China

**Keywords:** Skin, Histiocytosis, Immunoglobulin G, Immunoglobulin G4-related disease

## Abstract

**Objective:**

The authors investigated the expression of IgG4 and IgG in cutaneous Rosai-Dorfman Disease (CRDD) to further improve the understanding of this disease.

**Methods:**

The authors retrospectively reviewed the clinicopathological features of 23 CRDD patients. The authors diagnosed CRDD by the presence of emperipolesis and immunohistochemical (IHC) staining of histiocytes consisting of S-100(+)/CD68(+)/CD1a(-) cells. The expressions of IgG and IgG4 in cutaneous specimens were assessed by IHC (EnVision) and quantitatively calculated by a medical image analysis system.

**Results:**

All 23 patients, including 14 males and 9 females, were confirmed to have CRDD. Their ages ranged from 17 to 68 years (mean 47.91 ± 14.16). The most frequently affected skin regions were the face, followed by the trunk, ears, neck, limbs, and genitals. In 16 of these cases, the disease presented as a single lesion. IHC staining of sections showed that IgG was positive (≥ 10 cells/High-Power Field [HPF]) in 22 cases, while IgG4 was positive (≥ 10 cells/HPF) in 18 cases. Moreover, the IgG4/IgG proportion ranged from 1.7% to 85.7% (mean 29.50 ± 24.67%, median 18.4%) in the 18 cases.

**Study limitations:**

In the majority of studies, as well as in the current study, the design. RDD is a rare disease, so the sample size is small. In the next studies to come, the authors will expand the sample for multi-center verification and in-depth study.

**Conclusion:**

The positive rates of IgG4 and IgG and the IgG4/IgG ratio assessed through IHC staining may be important in understanding the pathogenesis of CRDD.

## Introduction

Rosai-Dorfman's disease (RDD) is a rare disease of non-malignant histiocytosis that used to be referred to as sinus histiocytosis with a large number of lymphadenopathy. It was named for Rosai and Dorfman, who established it as a new entity in two reports in 1969 and 1972.^1^, ^2^ Mutations in *KRAS* and *MAP2K1* may be related to the pathogenesis of RDD.^3^ RDD usually occurs in children and young adults, with a slight male predominance of 58% and a greater incidence among patients of African descent.^1^, ^4^ It is estimated that up to 43% of RDD patients have extranodal involvement, especially in the upper respiratory tract, skin, and bones.^5^ Cutaneous RDD (CRDD) is used to describe this disease when it is limited to the skin without systemic involvement.^1^, ^6^, ^7^ Histologically speaking, histiocytes are large and foamy with abundant cytoplasm and vesicular nuclei.^8^ Emperipolesis (histiocytes containing intact cells, especially lymphocytes and plasma cells) is a typical, but not unique, feature of RDD.^9^ The presence of S100, CD68-positive-stained, and CD1a-negative-stained histiocytes by Immunohistochemistry (IHC) is important for the diagnosis of RDD. Many other markers have also been reported to be positive in CRDD, such as CD14, CD15, CD163, and factor 13a.^8^ However, the expressions of IgG4 and IgG in CRDD have not been investigated. An incremental number of IgG4-positive plasma cells in skin lesions are believed to be related to IgG4-Related Disease (IgG4-RD). IgG4-RD is a chronic, inflammatory, systemic, and fibrotic disease that can affect almost every organ. There was a report that found that some RDD exhibited features of IgG4-RD, but the relevance between CRDD and IgG4-RD was not examined.^10^ To further investigate the relevance between CRDD and IgG4-RD, the authors evaluated the clinical manifestations and histopathological characteristics of 23 cases of CRDD and detected the expression of IgG and IgG4, which may provide novel biomarkers (IgG4, IgG, and the proportion of IgG4/IgG) for the identification of CRDD and help shed light on its underlying pathogenesis.

## Materials and methods

Twenty-three cases were collected from two different hospitals during the period from August 2009 to August 2018. All cases were diagnosed by 2 senior pathologists according to the latest WHO diagnostic criteria. This work was authorized by the ethics committee and conducted under the ethical principles of the Human Rights Declaration adopted in Helsinki and was in accordance with the Rules for Good Practice in the Clinical Study. The authors informed all patients of informed consent.

Skin biopsies were fixed in a buffered 10% formalin solution, processed, and embedded in paraffin. Paraffin blocks were cut into 4–5 μm sections using a microtome and placed on glass slides.

Hematoxylin-eosin (HE) was used for classical histopathology and IHC was performed on the same biological material embedded in paraffin. Each immunostaining reaction included a negative control (without incubation with the primary antibody). The IgG4 and IgG antibodies (ready to use) were bought from Zhongshan Golden Bridge Biological Technology Company.

IgG4(+) and IgG(+) cells were counted separately using an Olympus BX40 microscope and Image J 1.52a computer software. Three high-power fields (HPF) with the maximum number of positive cells were counted and an average number per HPF was counted. One HPF represents an area of 0.24 mm^2^.

## Results

### Clinical features

**Figure 1:**
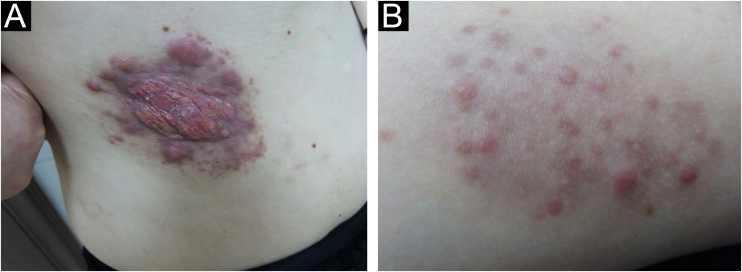
(A) Infiltrating plaque type: infiltrating yellow-red plaque, with uneven surface, and multiple dark red nodules of different sizes at the edge. (B) Nodular papule type: local distribution of multiple red papules of 0.2‒0.5 cm in size on the surface of pale erythema, showing isolated and non-fused shape

**Figure 2:**
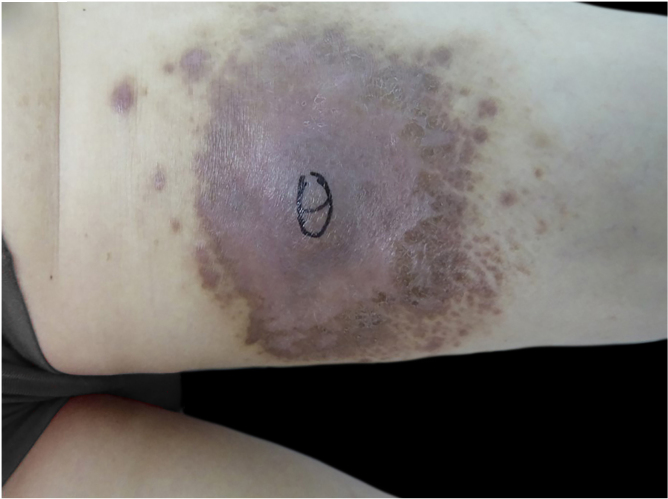
Tumor like type: Dark red palm-sized mass on flexor aspect of left thigh with obvious central uplift and multiple dark red nodules in the surrounding surface

The ages of the 23 patients with CRDD, including 14 males and 9 females, ranged from 17 to 68 years (mean 47.91 ± 14.16). Among them, 16 patients had single lesions, while the other 7 had multiple lesions. Ten patients presented with plaques, 12 patients presented with patches, and 1 patient presented with a sub-axilla mass. In most of the 23 cases of CRDD, the lesions were distributed on the face and chest. In 9 cases, the lesion was in another location such as a limb, back, or thigh. The clinical characteristics of all patients were described in Figs. 1 and 2 and summarized in Table 1.

**Table 1:** Demographic and clinical data

Case^a^	Age	Gender^b^	Duration^c^	Skin lesion description	Quantity	Pain	Comorbidity
1	57	M	20d	1 cm × 1 cm Upper right lip red plaque	1	No	None
2	23	F	6m	5 cm × 4 cm Upper right limb brown patch	1	No	None
3	53	M	1y	1 cm × 1 cm Right chest dull-red patch	1	No	None
4	59	F	1y	10 cm × 10 cm Right chest red plaque	1	No	None
5	28	F	6m	0.3 cm × 0.2 cm Right breast red papule	1	Yes	None
6	52	M	6m	15 cm × 10 cm Right lower back Red plaque	1	Yes	None
7	17	M	6y	3 cm × 2 cm Upper left limb red plaque	1	No	None
8	50	M	3m	3 cm × 2.5 cm Left cheek red patch	1	No	None
9	61	M	1y	Generalized skin eruption with red patch papule	many	No	None
10	57	F	6m	0.5 cm × 0.4 cm Right chest brown papule	1	No	None
11	38	F	2m	0.8 cm × 0.8 cm Left calf dull-red papule	1	No	None
12	57	M	4m	0.7 cm × 0.6 cm Left chest red papule	1	No	None
13	68	M	1y	2 cm × 1 cm Left temporal red plaque	1	No	None
14	61	M	1m	2 cm × 2 cm Plaque in chest and abdomen	3	No	None
15	63	F	2y	3 cm × 3 cm Left thigh dull-red patch	1	No	None
16	20	M	50d	1 cm × 1 cm Chest and abdomen plaques	5	No	None
17	59	F	7m	1 cm × 1 cm Left cheek red plaque	1	No	None
18	40	M	2y	3 cm × 3 cm Right thigh dark brown patch	1	No	None
19	54	M	2y	Generalized skin eruption with brown papules	1	No	None
20	39	F	2m	1 cm × 1.5 cm Left cheek red plaque	many	No	None
21	42	F	6m	2 cm × 1 cm Left cheek red plaque	2	No	None
22	52	M	6m	0.3 cm × 0.3 cm Right thigh papules	2	No	None
23	52	M	1y	3 cm × 3 cm Left sub-axillary mass	2	No	None

a Case 1 to case 12 were from Second Xiangya Hospital of Central South University and case 13 to case 23 were from Dermatology Hospital of Southern Medical University.

b M, Male; F, Female.

c y, Years; m, Months; d, Days.

### Pathologic features

**Figure 3:**
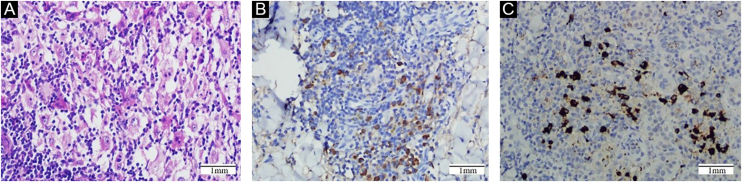
(A) It showed a diffused infiltration of large histiocytes, lymphocytes, and plasma cells with scattered neutrophils. (B) Expression of IgG. (C) Expression of IgG4

The authors observed diffused infiltration of large histiocytes, plasma cells with scattered neutrophils, and lymphocytes in all sections (Fig. 3A). In most cases, histocytes were large with wide and pale cytoplasm and evident rounded nuclei. However, the histocytes showed a foamy appearance or multinucleation in four cases. The authors found diffused fibrosis and collagen deposition without fibroblast infiltration in one case. Emperipolesis was found in most CRDD cases. Moreover, the authors observed red blood cells engulfed by histocytes in one case.

Immunohistochemical features were shown in Fig. 3B and C and were summarized in Table 2. The results revealed significant expression of IgG4 in 6 patients (> 50 cells/HPF), moderate expression of IgG4 in 3 patients (30–50 cells/HPF), mild expression in 9 patients (10–29 cells/HPF) and negative infiltration in 5 patients (< 10 cells/HPF). The IgG4/IgG ratio ranged from 1.7% to 85.7% (mean 29.5%, median 20.8%) in all positive cases, and the IgG4/IgG ratios in 7 cases were over 40%.

**Table 2:** Immunohistochemical features of samples

Case^a^	Features specific for RDD			Antibodies examined may contribute to diagnosis		
	S100^b^	CD68^b^	CD1a^b^	IgG4+/HPF^c^	IgG+/HPF^c^	IgG4/IgG%^c^
1	+	+	−	46	81	56.8%
2	+	+	−	33	40	82.5%
3	+	+	−	11	65	16.9%
4	+	+	−	35	90	38.9%
5	+	+	−	20	220	9.1%
6	+	+	−	101	213	47.4%
7	+	+	−	72	84	85.7%
8	+	+	−	10	265	3.8%
9	+	+	−	1	4	25%
10	+	+	−	9	49	18.4%
11	+	+	−	1	46	2.2%
12	+	+	−	12	12	13.3%
13	+	+	−	10	190	5.3%
14	+	+	−	71	160	44.3%
15	+	+	−	69	116	59.5%
16	+	+	−	19	79	24.1%
17	+	+	−	6	178	3.4%
18	+	+	−	18	182	9.9%
19	+	+	−	65	118	55.1%
20	+	+	−	2	120	1.7%
21	+	+	−	78	218	35.8%
22	+	+	−	10	48	20.8%
23	+	+	−	21	117	17.9%

a Case 1 to case 12 were from Second Xiangya Hospital of Central South University and case 13 to case 23 were from Dermatology Hospital of Southern Medical University.

b The immunohistochemical expression of S100, CD68 and CD1a were collected from existing pathologic reports.

c Three high-power fields (400×) with the maximum number of positive cells were counted, and an average number per HPF was calculated and presented in the Table 2.

## Discussion

Compared with classic systemic RDD, CRDD seems to occur more frequently in Asians and has a female predominance.^11^ The disease usually affects middle-aged people from 40 to 60 years. In the 23 patients included in this study, 65% were over 50 years, an age at which female predominance is less obvious.

In contrast to classic RDD with obvious systemic symptoms, CRDD is generally limited to the skin. Skin lesions of cutaneous RDD vary in morphology, size, color, and location. The clinical manifestations include papules, plaques, nodules, and pustules.^12^

Emperipolesis is a phenomenon of the lymphocytes in which they maintain their structure and function while entrapped in the cytoplasm of histiocytes. It is not a unique feature of CRDD and can also appear in Hodgkin lymphoma, malignant melanoma, and some other malignant tumors.^13^ Therefore, the most recognized diagnostic criteria of CRDD are positive IHC staining for CD68 and S-100 protein and negative staining for CD1a. Despite this, the diagnosis of RDD still remains to be a challenge: this is probably due to its overlapping clinical manifestations, morphology, and IHC features with other entities. RDD is likely to be confused with IgG4-RD in terms of clinical manifestations. The increase of IgG4^+^ plasma cell numbers has been found in 70 nodal and extranodal cases of RDD.^14^

The most recognized diagnostic standards for IgG4-RD based on its histology and IHC are as follows: (1) Diffuse or characteristic enlargement, tumors, nodules, or hypertrophy of one or more organs; (2) Serum IgG4 levels increased ≥1350 mg/L; and (3) Histopathology showing obvious lymphocyte and plasma cell infiltration and fibrosis, or the infiltration of IgG4-positive plasma cells with the ratio of IgG4/IgG-positive plasma cells being more than 40% and the quantity of IgG4-positive cells being more than 200/HPF in the skin.^15^ Zhang and colleagues speculated that IgG4-RD and RDD may share a common pathogenesis, or that IgG4-RD features may occur at some stage of RDD,^10^ which makes identification more difficult. In the present study, 4 patients who were diagnosed with CRDD also meet the diagnostic criteria of IgG4-RD, with ratios of IgG4/IgG positive plasma cells of more than 40%. These results showed that RDD and IgG4-RD were not completely distinct from each other. However, in the 23 total cases of CRDD, the quantity of IgG4-positive cells was less than 200/HPF. The authors can therefore determine whether the patient is RDD, IgG4-RD, or an overlap of the two through the IHC results of IgG, IgG4 and the ratio of IgG4/IgG plasma cells combined with the pathological manifestations and the staining results of s-100, CD68, and CD1a.

Generally, CRDD is a self-limiting and self-curing disease. However, in a minority of cases, the skin lesions present invasive growth that must be treated with excision, cryotherapy, methotrexate, retinoids, steroids, and/or thalidomide.

## Conclusion

Cutaneous RDD is a rare, nonmalignant disease characterized by certain clinical and pathological features. The presence of emperipolesis and IHC staining of histiocytes constituting S-100(+)/CD68(+)/CD1a(−) cells remains the main diagnostic criteria. Positive staining of IgG4 and IgG and the proportion of IgG4/IgG may be important for distinguishing RDD from IgG4-RD.

## Informed consent

Informed consent was obtained from all individual participants included in the study.

## Financial support

This study was supported by the National Natural Science Foundation of China (nº 81500187, nº 81502710).

## Authors' contributions

Puyu Zou: Contributed to the critical literature review and study concept and design; wrote the manuscript, and did the statistical analysis.

Yu Liu: Contributed to the critical literature review and study concept and design.

Yi Zhan: Conducted data collection, analysis and interpretation.

Xueru Zeng: Effectively participated in the research orientation, and the propaedeutic and therapeutic conduct of the studied cases.

Guiying Zhang: Made a manuscript critical review of the literature and finally approved the final version of the manuscript.

## Conflicts of interest

None declared.
